# The antagonistic pleiotropy of tau phosphorylation: plasma tau species in newborns and Alzheimer's disease

**DOI:** 10.1093/braincomms/fcag413

**Published:** 2026-10-29

**Authors:** Fernando Gonzalez-Ortiz, Bjørn-Eivind Kirsebom, Cristiano Santos, Burak Arslan, Julia K Gundersen, Hlin Kvartsberg, Karin Sävman, Michael Turton, Peter Harrison, Helena Sophia Coley Gleerup, Kristian Steen Frederiksen, Steen Gregers Hasselbalch, Anja Hviid Simonsen, Tormod Fladby, Helena Barreto Henriksson, Sofia Frandberg, Ann Hellström, Floris Groenendaal, Michel N Nafash, Ella N Klaus, Xuemei Zeng, Thomas K Karikari, Henrik Zetterberg, Ulrika Sjöbom

**Affiliations:** Department of Psychiatry and Neurochemistry, Institute of Neuroscience and Physiology, the Sahlgrenska Academy at the University of Gothenburg, Mölndal 431 80, Sweden; Royal College of Physicians of Edinburgh, Edinburgh EH2 1JQ, Scotland; Department of Neurology, University Hospital of North Norway, Tromsø 9038, Norway; Department of Psychology, Faculty of Health Sciences, The Arctic University of Norway, Tromsø 9037, Norway; Department of Neurology, Akershus University Hospital, Lørenskog 1478, Norway; Department of Psychiatry and Neurochemistry, Institute of Neuroscience and Physiology, the Sahlgrenska Academy at the University of Gothenburg, Mölndal 431 80, Sweden; Department of Psychiatry and Neurochemistry, Institute of Neuroscience and Physiology, the Sahlgrenska Academy at the University of Gothenburg, Mölndal 431 80, Sweden; Department of Neurology, Akershus University Hospital, Lørenskog 1478, Norway; Clinical Neurochemistry Laboratory, Sahlgrenska University Hospital, Mölndal 431 80, Sweden; Department of Paediatrics, Institution of Clinical Sciences, Sahlgrenska Academy, University of Gothenburg, Gothenburg 405 30, Sweden; Bioventix Plc, Surrey GU9 7SX, UK; Bioventix Plc, Surrey GU9 7SX, UK; Danish Dementia Research Centre, Department of Neurology, Copenhagen University Hospital, Rigshospitalet, Copenhagen 2100, Denmark; Danish Dementia Research Centre, Department of Neurology, Copenhagen University Hospital, Rigshospitalet, Copenhagen 2100, Denmark; Department of Clinical Medicine, Faculty of Health and Medical Sciences, University of Copenhagen, Copenhagen 2200, Denmark; Danish Dementia Research Centre, Department of Neurology, Copenhagen University Hospital, Rigshospitalet, Copenhagen 2100, Denmark; Department of Clinical Medicine, Faculty of Health and Medical Sciences, University of Copenhagen, Copenhagen 2200, Denmark; Department of Clinical Medicine, Faculty of Health and Medical Sciences, University of Copenhagen, Copenhagen 2200, Denmark; Department of Neurology, Akershus University Hospital, Lørenskog 1478, Norway; Institute for Clinical Medicine, University of Oslo, Oslo 1478, Norway; Department of Clinical Neuroscience at the Institution of Neuroscience and Physiology, Sahlgrenska Academy at University of Gothenburg, Gothenburg 405 30, Sweden; Department of Laboratory Medicine, Institute of Biomedicine at Sahlgrenska Academy at University of Gothenburg, Gothenburg 405 30, Sweden; Department of Laboratory Medicine, Institute of Biomedicine at Sahlgrenska Academy at University of Gothenburg, Gothenburg 405 30, Sweden; Department of Clinical Neuroscience at the Institution of Neuroscience and Physiology, Sahlgrenska Academy at University of Gothenburg, Gothenburg 405 30, Sweden; Department of Ophthalmology, Sahlgrenska University Hospital, Gothenburg 413 45, Sweden; Department of Neonatology, Wilhelmina Children’s Hospital, University Medical Center Utrecht and Utrecht University, Utrecht 3584 CX, Netherlands; Department of Psychiatry, School of Medicine, University of Pittsburgh, Pittsburgh, USA; Department of Psychiatry, School of Medicine, University of Pittsburgh, Pittsburgh, USA; Department of Psychiatry, School of Medicine, University of Pittsburgh, Pittsburgh, USA; Biofluid Biomarker Laboratory, Western Psychiatric Hospital, University of Pittsburgh Medical Center, Pittsburgh 15213, USA; Alzheimer's Disease Research Center, University of Pittsburgh, Pittsburgh 15213, PA, USA; Department of Psychiatry, School of Medicine, University of Pittsburgh, Pittsburgh, USA; Biofluid Biomarker Laboratory, Western Psychiatric Hospital, University of Pittsburgh Medical Center, Pittsburgh 15213, USA; Alzheimer's Disease Research Center, University of Pittsburgh, Pittsburgh 15213, PA, USA; Department of Psychiatry and Neurochemistry, Institute of Neuroscience and Physiology, the Sahlgrenska Academy at the University of Gothenburg, Mölndal 431 80, Sweden; Clinical Neurochemistry Laboratory, Sahlgrenska University Hospital, Mölndal 431 80, Sweden; Department of Neurodegenerative Disease, UCL Institute of Neurology, Queen Square WC1N 3BG, London, UK; UK Dementia Research Institute at UCL, London WC1N 3BG, UK; Department of Pathology and Laboratory Medicine, University of Wisconsin School of Medicine and Public Health, Madison, WI 53792, USA; Wisconsin Alzheimer’s Disease Research Center, University of Wisconsin School of Medicine and Public Health, University of Wisconsin-Madison, Madison, WI 53792, USA; Centre for Brain Research, Indian Institute of Science, Bangalore 560012, India; Department of Clinical Neuroscience at the Institution of Neuroscience and Physiology, Sahlgrenska Academy at University of Gothenburg, Gothenburg 405 30, Sweden; Department of Ophthalmology, Sahlgrenska University Hospital, Gothenburg 413 45, Sweden; Institute of Health and Care Sciences, Sahlgrenska Academy, University of Gothenburg, Gothenburg 405 30, Sweden

**Keywords:** plasma phospho-tau, babies, Alzheimer’s disease, neurodegeneration, neurodevelopment

## Abstract

Tau phosphorylation is essential for early brain development but is also central to Alzheimer’s disease pathogenesis, suggesting an antagonistic pleiotropy model in which the same biology is beneficial early in life and harmful in ageing. Here, we investigate whether healthy term newborns exhibit higher concentrations of multiple phosphorylated tau species in plasma and compare their profiles against older adults and Alzheimer’s disease patients. In this multicentre study, we used the Simoa HD-X platform to measure plasma phospho-tau (p-tau) species (p-tau217 and p-tau205), Glial fibrillary acidic protein (GFAP), Neurofilament light (NfL) and amyloid-β species (Aβ1–40 and Aβ1–42) in healthy term newborns (*n* = 42), aged controls (*n* = 41) and patients with Alzheimer’s disease (*n* = 73). Perinatal associations were examined using multivariable regression adjusted for gestational age and birth weight. In an exploratory subset analysis comprising 12 aged controls and 27 term newborns, 220 analytes were measured using the Central Nervous System panel on the NULISA proteomics platform. Group comparisons were performed using either linear regression or Welch *t*-tests with Benjamini–Hochberg correction for multiple testing. In the Simoa analysis, plasma p-tau217 and p-tau205 showed a stepwise increase from controls to Alzheimer’s disease and from Alzheimer’s disease to newborns. Mean p-tau217 concentrations were 2.6 pg/mL in controls, 8.6 pg/mL in Alzheimer’s disease and 17 pg/mL in newborns; corresponding p-tau205 concentrations were 0.5, 0.7 and 3.3 pg/mL. All pairwise differences for both tau species were significant. Correlations between p-tau markers differed across groups. Newborns showed a strong correlation (*r* = 0.81, *P* < 0.001), whereas correlations were weaker in Alzheimer’s disease (*r* = 0.44, *P* < 0.001). Newborns also had the highest GFAP concentrations, whereas NfL was highest in Alzheimer’s disease and lowest in newborns. Mode of delivery affected GFAP and NfL levels, but not p-tau217 or p-tau205, arguing against birth stress as the main driver of neonatal tau phosphorylation. The NULISA analysis independently supported broad neonatal enrichment of tau. Phosphorylated tau species were markedly higher in newborns than controls, and multiple epitopes, including the brain-derived forms of p-tau181, p-tau205, p-tau212, p-tau217 and p-tau231, clustered among proteins with positive newborn-control differences and strong statistical significance. Our findings identify a high early-life phospho-tau state across multiple epitopes that exceeds Alzheimer’s disease levels but occurs outside of the amyloid/neurodegenerative biomarker profile. The results support the hypothesis that tau phosphorylation has divergent biological meanings across the lifespan and motivate mechanistic studies of how the developing brain tolerates high phospho-tau without an amyloid/neurodegenerative biomarker profile.

## Teaser

Gonzalez-Ortiz F. *et al*. report increased plasma levels of multiple phospho-tau species (181, 205, 212, 217 and 231) and glial fibrillary acidic protein in newborns compared with adults and Alzheimer’s disease patients, while neurofilament light and amyloid-β show significantly lower concentrations.

## Graphical Abstract

**Graphical Abstract:**
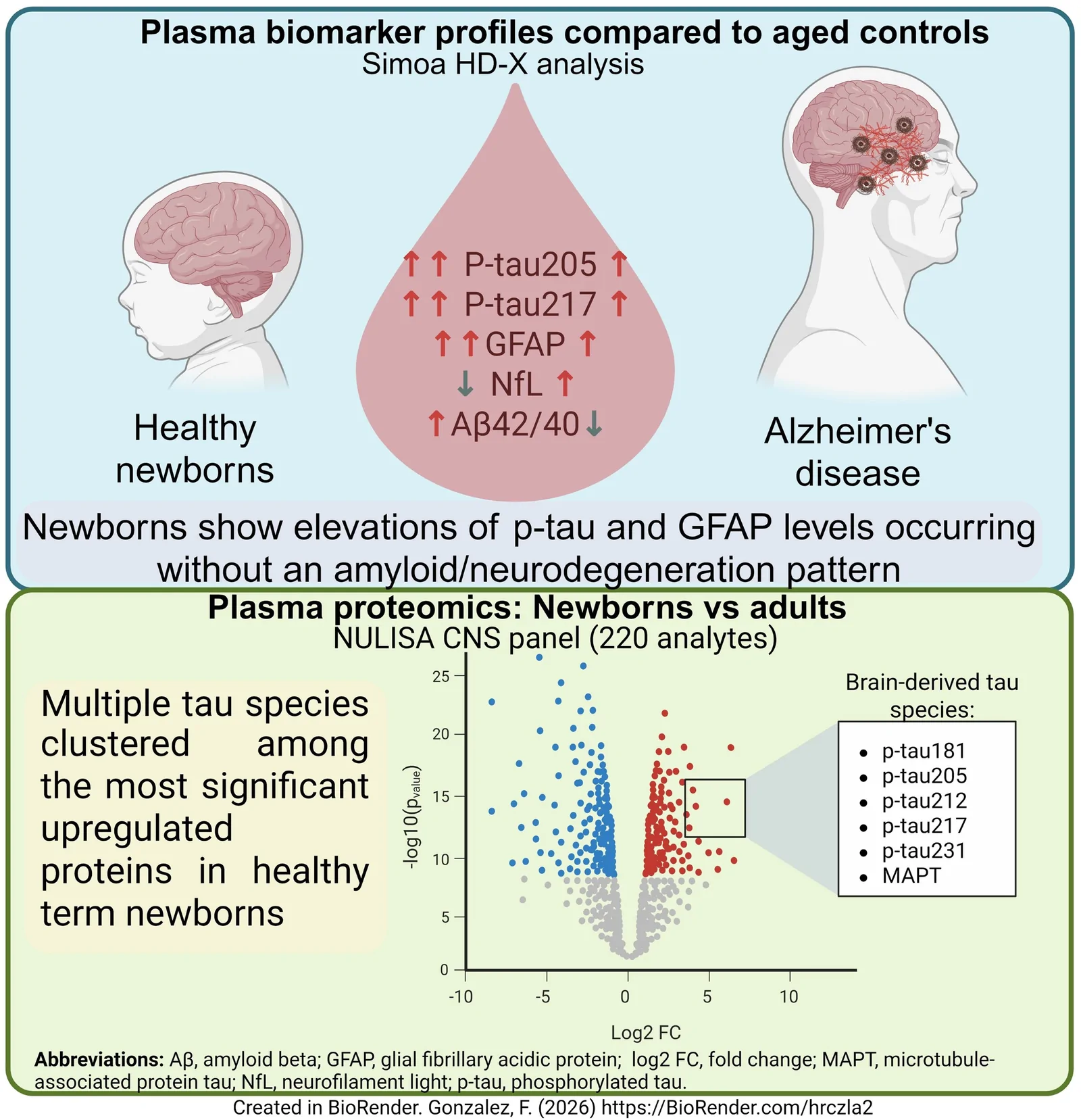

## Introduction

Tau is a microtubule-associated protein that plays a central role in both the development and pathology of the human brain.^1,2^ During early development, tau phosphorylation supports axonal growth, cytoskeletal organization and synaptic maturation, processes indispensable for establishing functional neural networks.^3,4^ In contrast, in late life, aberrant phosphorylation promotes tau aggregation and tangle formation that leads to a neurodegenerative cascade of events characteristic of Alzheimer’s disease, the most common cause of dementia around the world.^5,6^ The concept of processes, in most cases mutations, that are beneficial in early life but pathological in the older population is known as ‘antagonistic pleiotropy’.^7^ Classic examples include the sickle cell mutation in haemoglobin β, which confers protection against severe malaria early in life but predisposes carriers to vaso-occlusive crises and anaemia later on, and the APOE ɛ4 allele, which is associated with decreased risk of spontaneous abortion and provides resilience against infections and malnutrition during youth while increasing Alzheimer’s disease risk in later life.^8-10^

While tau hyperphosphorylation can be framed as an example of antagonistic pleiotropy, phosphorylated tau (p-tau) biomarkers have mostly been studied in the context of Alzheimer’s disease, with the physiological relevance of these biomarkers during early human development remaining largely unexplored.^2,11,12^ For example, in the 2024 Revised Criteria for Alzheimer’s diagnosis, p-tau biomarkers are stratified into Core 1 (early changing, disease-defining, e.g. p-tau217) and Core 2 (later-stage, e.g. p-tau205) according to when they deviate in the Alzheimer’s disease continuum.^13^ The 2024 criteria consider abnormalities in CSF or plasma p-tau181, p-tau217, and p-tau231 sufficient to establish a diagnosis of Alzheimer’s disease, suggesting that these biomarkers have pathognomonic value.^13^

In our previous study, p-tau217 levels in umbilical cord plasma from healthy newborns were found to be 10- to 12-fold higher than in adult controls and 2- to 3-fold higher than in Alzheimer’s disease, with a rapid decline during the first months of life after birth.^11^ This temporal profile is consistent with a regulated early-life process of phosphorylated tau production, release and clearance in the developing and neonatal brain.^11^ Whether such elevations reflect intrinsic neurodevelopmental processes or extrinsic perinatal stress responses remain under debate.^14,15^ It has been proposed that the surge in neonatal p-tau217 may partly reflect transient ‘synaptic stress remodeling’ induced by the physiological challenges of birth, including mechanical strain, hypoxia and haemodynamic fluctuations.^15^ This hypothesis aligns with findings that acute neuronal stress, such as ischaemia or cardiac arrest, transiently increases plasma p-tau species (e.g. p-tau231 and p-tau217) without leading to chronic pathology.^16,17^ Thus, p-tau may serve as a marker of synaptic remodelling and stress resilience, bridging developmental and degenerative contexts.^2,18^

Leveraging the antagonistic pleiotropy of tau phosphorylation could open new therapeutic avenues aimed at modulating tau function in different tauopathies.^19^ Similar conceptual frameworks have been successfully applied in other disease contexts; for instance, Lin *et al*. demonstrated that drug-induced antagonistic pleiotropy in cancer can be harnessed to design ‘evolutionary traps’ that selectively eliminate resistant tumour populations.^20^ Analogously, understanding the pleiotropic balance of tau phosphorylation could enable the rational design of interventions that preserve its developmental benefits while preventing its pathological consequences.^19,21^

Building on prior evidence that cord blood p-tau217 is markedly elevated in newborns, we aim to investigate whether this phenomenon extends to other phospho-tau species, whether it occurs within an Alzheimer’s disease-like glial, axonal and amyloid biomarker context and whether it is explained by mode of delivery.^11^ In this study, we directly compared plasma biomarker profiles across the human lifespan, spanning healthy newborns, aged controls and individuals with Alzheimer’s disease, using the 2024 Alzheimer’s Association-recommended biomarker panel (p-tau217, p-tau205, GFAP and NfL), with particular emphasis on phosphorylated tau species.^13^ Additionally, we also performed an exploratory subset analysis using the NULISA CNS panel, which enabled simultaneous assessment of multiple tau species and additional central nervous system-enriched proteins in newborns and aged controls.^22^ Altogether, we aimed to (i) compare plasma biomarker profiles across developmental and degenerative contexts, with particular focus on how early-life phospho-tau profiles differ from those observed in Alzheimer’s disease; (ii) investigate whether perinatal factors, including mode of delivery, gestational age, birth weight and sex are associated with neonatal biomarker levels; and (iii) determine whether the neonatal phospho-tau signal is supported by an independent multiplex platform. Accordingly, these data should be interpreted as hypothesis-generating rather than mechanistically definitive.

## Material and methods

### Cohort characteristics

#### Sahlgrenska University Hospital plasma newborns cohort (cohort-1)

The cohort consists of cord blood samples from 42 term-born newborns collected for the National Swedish Cord Blood Bank (Gothenburg, Sweden). The samples were collected for the purpose of producing stem cell graft for transplantation and to investigate biomarkers related to the number of those cells. The mean (SD) gestational age at birth was 39.7 (1) weeks, and the mean birth weight was 3747.9 (460.3) g. A total of 23 infants (56%) were delivered by caesarean section (C-section), of which one was emergency and the remaining were planned. In addition, 22 (53.7%) of the newborns were females (one infant lacked information on mode of delivery, sex and other perinatal factors).

As part of the inclusion criteria, cord blood was only collected from newborns with healthy mothers and uncomplicated/normal pregnancies and that a thorough medical history was taken from the parents through interviews before the collection of the cord blood to exclude hereditary metabolic or haematopoietic and other malignant diseases. Ethical approval was obtained from the Swedish Ethical Review Authority (no. 916–11). Demographic and biomarker information is provided in Table 1.

**Table 1:** Descriptive data for cohort-1 and -2

	Aged controls *n* = 41	AD *n* = 73	Newborns *n* = 42
**Age** Mean (SD) [missing]	†61.7 (10.9)	†69.8 (8.9)	**‡**39.7 (1.0) [1]
**Female** *n* (%) [missing]	23 (56%)	49 (67%)	22 (54%) [1]
**MMSE** median (IQR) [missing]	30.0 (1.0) [3]	24.0 (6) [4]	§
**Delivery mode** *n* (%) [missing]			
C-section	§	§	23 (56%) [1]
Vaginal	§	§	18 (44%) [1]
**Birth weight Gr.** Mean (SD)	§	§	3747.9 (460.3) [1]
**Plasma P-tau217 pg/mL** Mean (SD)	2.6 (1.4)	8.6 (5.8)	17.0 (7.4)
**Plasma P-tau205 pg/mL** Mean (SD)	0.45 (0.27)	0.70 (0.81)	3.28 (1.90)
**Plasma GFAP pg/mL** Mean (SD)	105.2 (80.6)	186.1 (75.7)	339.7 (214.9)
**Plasma NfL pg/mL** Mean (SD)	16.3 (11.8)	25.3 (20.3)	11.6 (14.4)
**Plasma Aβ1–40 pg/mL** Mean (SD)	93.6 (21.2)	101.2 (34.8)	21.1 (11.5)
**Plasma Aβ1–42 pg/mL** Mean (SD)	5.6 (1.3)	5.2 (2.0)	3.9 (2.0)

Abbreviations: AD, Alzheimer’ disease; MMSE, Mini Mental Status Examination; SD, standard deviation; *n*, number of cases; %, percentage; IQR, Interquartile range; Gr, Gram; †, Age in years; **‡**, Gestational age in weeks; §, Not applicable.

#### Danish Dementia Research Centre plasma cohort (cohort-2)

Cohort-2 consists of 114 patients (41 controls and 73 Alzheimer’s disease) referred to the Danish Dementia Research Centre (DDRC) in Copenhagen. The catchment area is the greater Copenhagen area. The patients have been referred for diagnostic evaluation of dementia either by general practitioners or specialist doctors. All patients had their medical history recorded, and cognitive tests including Mini-Mental State Examination (MMSE) and CSF biomarker profiles. Patient diagnostic assessments were evaluated by a panel consisting of neurologists, geriatricians, neuropsychologists and nurses and a consensus conference for the determination of the cognitive syndrome and aetiological diagnosis. For a more detailed description of the diagnostic procedure ,see Hasselbalch *et al.* 2025.^23^ Inclusion criteria included available CSF and plasma sample and informed consent for the Danish Dementia Biobank. Patients were excluded if their lumbar puncture was done as part of a clinical drug trial. The study was approved by the ethical committees of the Capital Region of Denmark, and the Danish Data Protection Agency. All biobank/database participants provided written informed consent for future research use and publication, and storage is approved by the Danish Data Protection Agency (P-2020-235). Demographic and biomarker information is provided in Table 1.

#### Biomarker analysis

The analysis of plasma biomarkers on Quanterix Simoa HD-X was performed at the University of Gothenburg in Sweden using the platform. Simoa plasma p-tau217 was measured using previously validated in-house methods, while plasma p-tau205 was measured using commercially available kits (item: #105634).^24^ Simoa plasma Aβ40, Aβ42, GFAP and NfL were measured using commercially available kits (Neuro4-plex E item: #103670). The NULISA analysis was performed at the University of Pittsburgh in Pittsburgh Pennsylvania using the commercially available CNS-panel.^22^ The CNS-panel included 220 analytes, among them brain-derived tau, p-tau181, p-tau205, p-tau212, p-tau217 and p-tau231 that were the primary focus of our analysis due to their strong association with Alzheimer’s disease pathology previously reported.^25-30^ The NULISA analysis was performed on a subset of available citrate plasma samples from cohort-1 (*n* = 27, Supplementary Table 1) and anonymous citrate plasma samples from adults with no clinical data available (*n* = 12), obtained from adults at Sahlgrenska University Hospital in Mölndal, Sweden, as reported in a previous publication.^31^

The newborns samples in cohort-1 were citrate plasma while the aged control and Alzheimer’s disease groups in cohort-2 included EDTA plasma samples. To derive an appropriate correction factor for the newborns’ citrate plasma samples, mean concentrations of all biomarkers were calculated in the subset of controls with paired EDTA and citrate samples (*n* = 8), and the ratios between EDTA and citrate means were computed. These ratios were then applied as correction factors to the newborns’ raw citrate plasma concentrations. See Supplementary Table 2.

#### Statistical analysis

All statistical analyses were performed in R (version 4.3.2, RStudio). For all Simoa-based biomarker analyses, plasma concentrations were log-transformed and subsequently z-standardized to reduce skewness and place effects on a comparable scale. For between-group comparisons, we fitted linear regression models with biomarker z-scores as the dependent variable and diagnostic group as the independent variable, using the control group as the reference category. From these models, we derived a set of prespecified pairwise contrasts between groups. These standardized group differences (β) are reported together with their corresponding *P*-values, which were adjusted for multiple comparisons on a per-biomarker basis (three contrasts per biomarker) using the Benjamini–Hochberg (BH) false discovery rate (FDR) procedure, reflecting our definition of each biomarker as a distinct biological outcome and the three prespecified pairwise contrasts as multiple comparisons of that outcome. In addition, fold changes, based on raw concentrations, were calculated for all diagnostic groups (Alzheimer’s disease and newborns) using the aged control group as the reference; for each biomarker and group, fold change was defined as the ratio of the mean concentration in that group to the mean concentration in the aged control group. To assess whether mode of delivery was associated with neonatal biomarker levels while accounting for other perinatal factors, we fitted multiple linear regression models with log-transformed and z-standardized biomarker values as the dependent variables and delivery mode as the independent variable, adjusting for gestational age and birth weight. Sex was also examined as a predictor in the perinatal regression analyses. Variance inflation factors (VIFs) were inspected for all models, and no evidence of collinearity among predictors was observed (all VIFs < 2). For these perinatal analyses, *P*-values were corrected for multiple testing using the BH method. Significance symbols in the figures correspond to BH-adjusted *P*-values (* *P* < 0.05, ** *P* < 0.01, *** *P* < 0.001). For all regression models, linear regression assumptions were evaluated by visual inspection of residual diagnostic plots. For analyses of perinatal factors, one C-section case with an extreme plasma NfL value was excluded from the NfL regression model because it was highly influential. Additionally, we examined associations between biomarkers by computing pairwise Spearman rank correlations within each diagnostic group. For each group and biomarker panel, *P*-values for the correlations were adjusted for multiple testing using the BH method. Finally, group differences in NULISA Protein Quantification (NPQ) values between newborns and aged controls were examined separately for each analyte using Welch’s two-sample *t*-tests, which do not assume equal variances between groups. NPQ values were analysed on their native log2 scale, and effect sizes were reported as mean log2 fold change, calculated as mean NPQ in newborns minus mean NPQ in aged controls. To account for multiple testing across analytes, *P*-values were adjusted using the BH FDR procedure. Because of the modest sample size, particularly in the aged control group, uncertainty around the mean log2 fold change was estimated using bootstrap 95% confidence intervals.

## Results

### Plasma p-tau205 and p-tau217 in healthy newborns, aged adults and Alzheimer's disease

Plasma p-tau205 and p-tau217 showed stepwise increases from controls to Alzheimer’s disease and from Alzheimer’s disease to newborns (Fig. 1). However, biomarker increases were more pronounced for p-tau217 in the different groups. Mean plasma p-tau217 concentrations were 2.6 ± 1.4 pg/mL in controls, 8.6 ± 5.8 pg/mL in Alzheimer’s disease and 17 ± 7.4 pg/mL in newborns. On the other hand, plasma p-tau205 followed a similar pattern, with means of 0.45 ± 0.27, 0.70 ± 0.81 and 3.28 ± 1.90 pg/mL, respectively. In Alzheimer’s disease cases compared with controls, plasma p-tau217 was markedly elevated (β = 1.31, *P* < 0.001, fold change = 3.29), whereas p-tau205 showed only a modest change (β = 0.29, *P* < 0.05, fold change = 1.55). In newborns compared with controls, both plasma p-tau217 (β = 2.07, *P* < 0.001, fold change = 6.50) and p-tau205 (β = 1.90, *P* < 0.001, fold change = 7.2) were strongly elevated compared with controls (Fig. 2). Importantly, while the correlation between p-tau205 and p-tau217 was strong in the newborns group (Spearman r = 0.81, *P* < 0.001, Supplementary Fig. 1), the correlation in the Alzheimer’s disease group was only moderate (Spearman *r* = 0.44, *P* < 0.001, Supplementary Fig. 1). Together, these findings suggest that the neonatal plasma phospho-tau profile spans evenly across multiple epitopes while in adults this relationship is different, supporting the concept proposed in the 2024 Framework with p-tau205 and p-tau217 reflecting different aspects of tau pathology in Alzheimer’s disease.^32^

**Figure 1:**
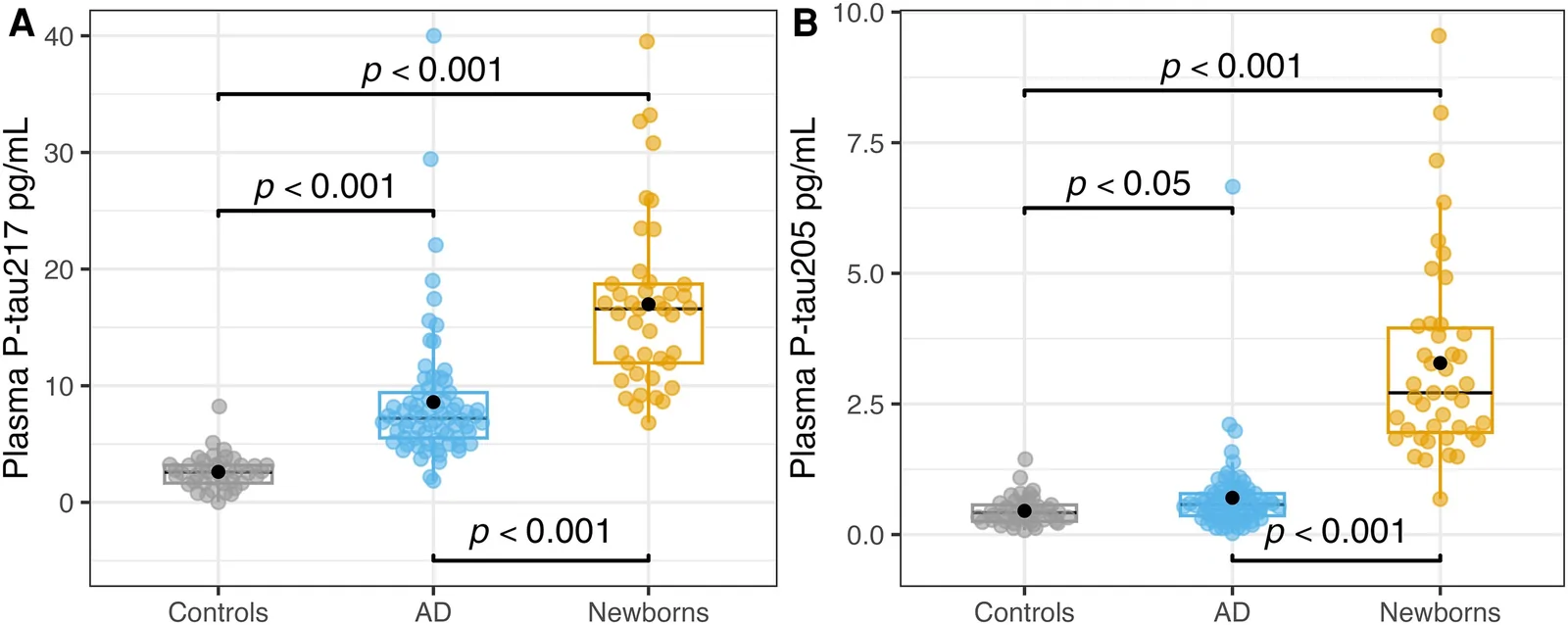
**Plasma Alzheimer’s disease-related biomarkers in adults with Alzheimer’s disease, controls and newborns.** Scatter-box plots show plasma p-tau217 (Panel **A**) and plasma p-tau205 (Panel **B**) for three pairwise group comparisons: Alzheimer’s disease (*n* = 73) versus controls (*n* = 41; *n* = 114), newborns (*n* = 42) versus controls (*n* = 83), and newborns versus Alzheimer’s disease (*n* = 115). Each dot represents an individual participant. Boxes indicate the interquartile range, horizontal lines within boxes indicate the median, whiskers indicate the spread of the data and black dots indicate the mean. Group differences were tested using linear regression models with z-scores of log-transformed biomarker concentrations as the dependent variable and diagnostic group as the independent variable, followed by prespecified pairwise contrasts. For both biomarkers, plasma concentrations were lowest in controls, higher in Alzheimer’s disease and highest in newborns. All pairwise group comparisons were statistically significant (*P* < 0.05). Abbreviations: p-tau, phosphorylated tau.

**Figure 2:**
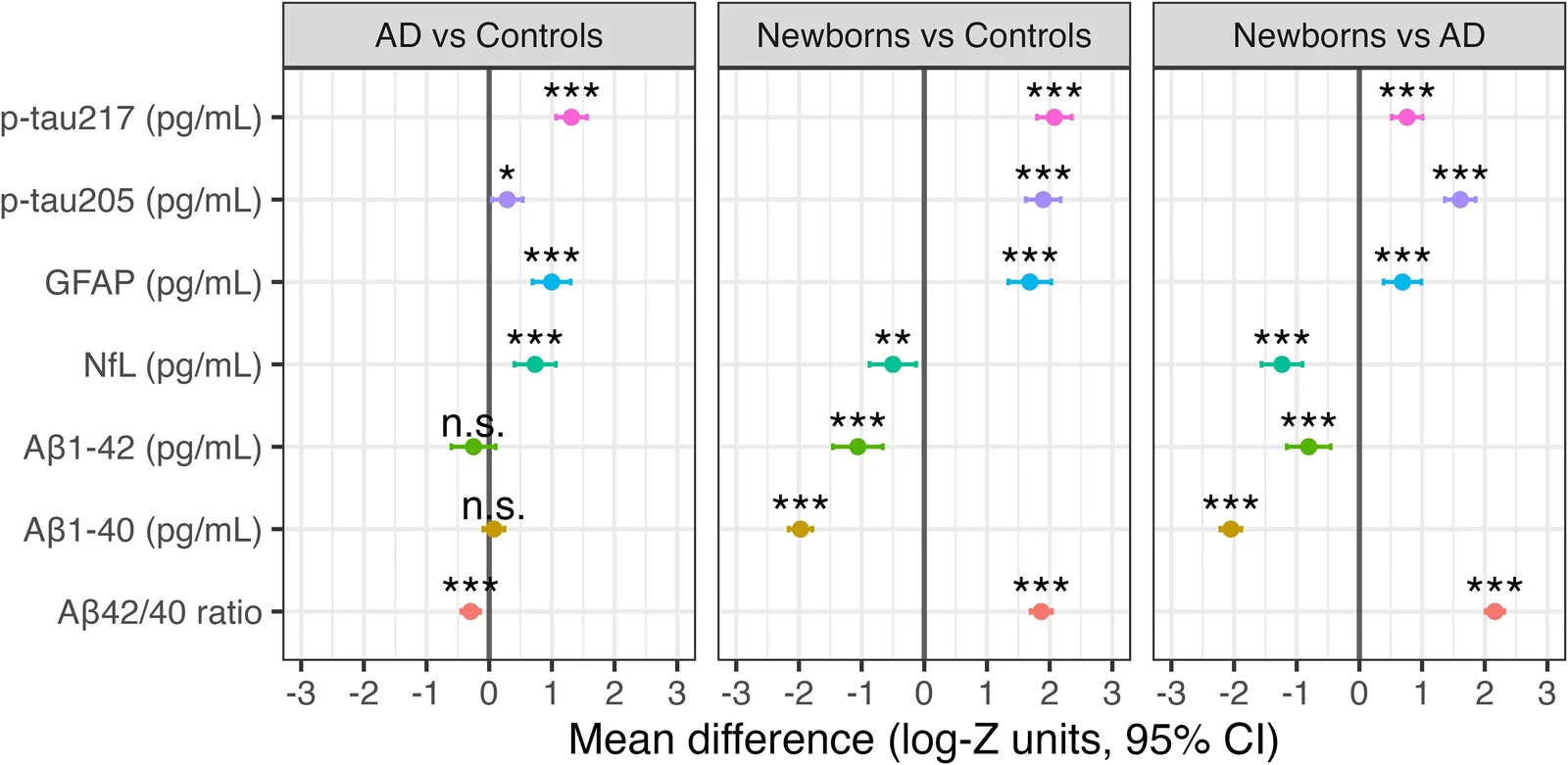
**Plasma Alzheimer’s disease-related biomarkers in adults with Alzheimer’s disease, cognitively unimpaired controls and newborns.** Forest plots show standardized group differences (β, in SD units; 95% CI) in plasma concentrations of p-tau217, p-tau205, GFAP, NfL, Aβ1–42, Aβ1–40 and the Aβ42/40 ratio for three pairwise group comparisons: Alzheimer’s disease (*n* = 73) versus controls (*n* = 41; *N* = 114), newborns (*n* = 42) versus controls (*n* = 83), and newborns versus Alzheimer’s disease (*n* = 115). Group differences were estimated using linear regression models with log-transformed biomarker z-scores as the dependent variable and diagnostic group as the independent variable, followed by prespecified pairwise contrasts. Positive values indicate higher concentrations in the group named first in each contrast. Asterisks denote the level of statistical significance for the group comparison (* adjusted *P* < 0.05; ** < 0.01; *** < 0.001), and ‘n.s.’ indicates non-significant differences. Abbreviations: SD, standard deviation; CI, confidence interval; p-tau, phosphorylated tau; GFAP, glial fibrillary acidic protein; NfL, neurofilament light chain; Aβ, amyloid-beta; n.s., not significant.

### Plasma NfL, GFAP and amyloid species in newborns, aged adults and Alzheimer’s disease

Glial and axonal injury markers showed group-dependent and partially divergent patterns (Fig. 2). In Alzheimer’s disease cases compared with aged controls, GFAP (β = 0.99, *P* < 0.001, fold change = 1.77) and NfL (β = 0.73, *P* < 0.001, fold change = 1.55) were elevated, and Aβ42/40 ratios were reduced (β = −0.30, *P* < 0.001), whereas differences in Aβ1–42 (β = −0.25) and Aβ1–40 (β = 0.08) were not significant. In newborns, GFAP showed an even larger increase relative to aged controls (β = 1.68, *P* < 0.001, fold change = 3.23), but in contrast to Alzheimer’s disease, NfL (β = −0.50, *P* < 0.01, fold change = 0.72), Aβ1–42 (β = −1.06, *P* < 0.001, fold change = 0.70) and especially Aβ1–40 (β = −1.97, *P* < 0.001 fold change = 0.23) were lower. Because the reduction in Aβ1–40 exceeded that of Aβ1–42, the Aβ42/40 ratio was increased (β = 2.16, *P* < 0.001, fold change = 3.11). Directly comparing newborns with Alzheimer’s disease cases, newborns showed higher GFAP (β = 0.69, *P* < 0.001), lower NfL (β = −1.23, *P* < 0.001), lower Aβ1–42 (β = −0.81, *P* < 0.001) and Aβ1–40 (β = −2.05, *P* < 0.001) and higher Aβ42/40 ratios (β = 2.17, *P* < 0.001). Although GFAP was elevated in newborns and Alzheimer’s disease, newborns differed from Alzheimer’s disease by showing lower NfL, lower absolute Aβ concentrations and a higher Aβ42/40 ratio, consistent with a different biological context.

### Association of plasma biomarkers with perinatal factors

To examine whether birth-related stress contributes to the neonatal biomarker profile, we compared newborns delivered by C-section (*n* = 23) with those delivered vaginally (*n* = 18). Because such differences may also be influenced by other perinatal factors, including gestational age and birth weight, we applied multiple linear regression models using log-transformed and z-standardized biomarker values (Fig. 3). VIFs were inspected for all models, and no evidence of collinearity among predictors was observed (all VIFs < 2). Group differences were evaluated while adjusting for gestational age and birth weight, and *P*-values were corrected for multiple testing using the BH FDR procedure. Vaginal delivery was similarly associated with higher GFAP (β = 1.06, *P* < 0.001) and NfL (β = 0.83, *P* < 0.05) concentrations. In contrast, plasma p-tau217, p-tau205, Aβ1–40 and Aβ1–42 were not significantly influenced by the mode of delivery (all *P* > 0.05). Higher gestational age was also associated with higher NfL (β = 0.39, *P* < 0.05) concentrations, whereas higher birth weight was associated with lower NfL (β = −0.35, *P* < 0.05). We did not observe any significant sex differences for the included biomarkers (all *P* > 0.05). These data suggest that the mode of delivery alone does not account for the neonatal plasma p-tau signal. However, they do not exclude contributions from other perinatal stressors or developmental factors.^33^ Please note that one C-section case was excluded from the NfL regression model because regression diagnostics indicated substantial influence (standardized residual = 4.62; Cook’s distance = 0.40), leaving 22 C-section and 18 vaginal-delivery cases in the primary NfL analysis (*n* = 40). In sensitivity analyses retaining the influential NfL observation, associations remained in the same direction but were attenuated: mode of delivery (β = 0.47, *P* = 0.189), gestational age (β = 0.23, *P* = 0.259) and birth weight (β = −0.24, *P* = 0.100). As before, sex remained non-significant (β = 0.21, *P* = 0.199).

**Figure 3:**
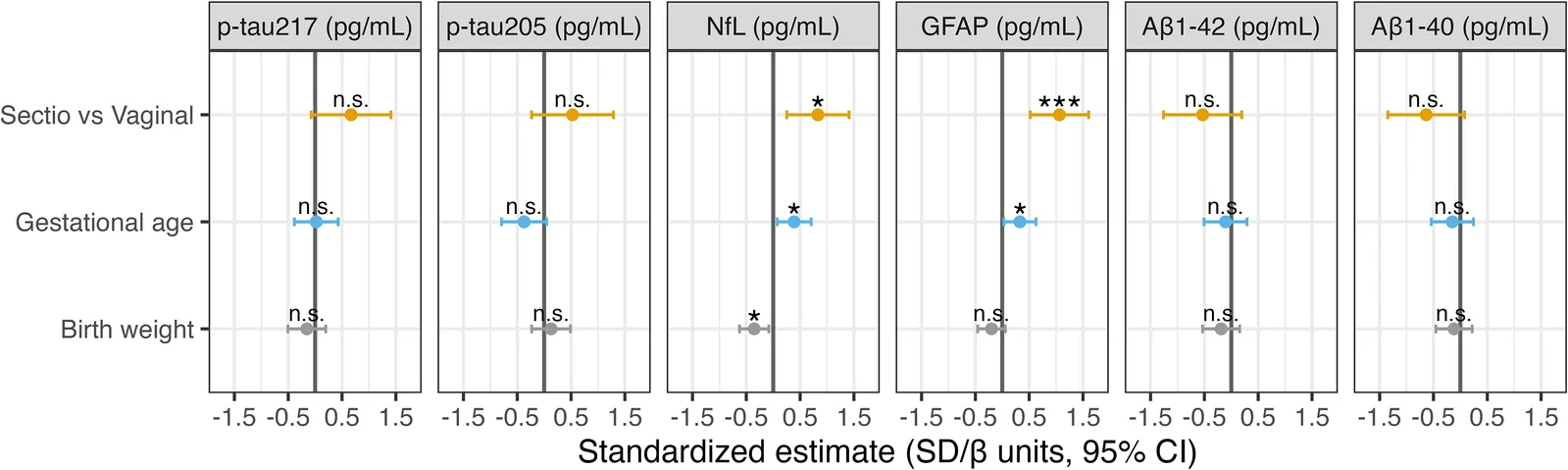
**Association between mode of delivery and newborn plasma Alzheimer’s disease-related biomarkers.** Forest plots show standardized regression coefficients (β, in SD units) with 95% confidence intervals from multivariable linear regression models in newborns with complete delivery-mode data (*n* = 41), with each plasma biomarker (log-transformed and z-standardized) as the outcome and mode of delivery (vaginal versus C-section), gestational age and birth weight as predictors. For the NfL model, one influential observation was excluded based on regression diagnostics (*n* = 40). The vertical line indicates no association (β = 0). *P*-values were adjusted for multiple testing using the Benjamini–Hochberg false discovery rate procedure; asterisks denote significant associations (* adjusted *P* < 0.05; ** < 0.01; *** < 0.001) and ‘n.s.’ indicates non-significant associations. Abbreviations: SD, standard deviation; NfL, neurofilament light chain; n.s., not significant.

### NULISA CNS panel analysis shows increased plasma p-tau181, p-tau205, p-tau212, p-tau217 and p-tau231 in newborns

We next used the NULISA CNS panel in a subset of plasma samples (newborns *n* = 27 and controls *n* = 12) to test whether the neonatal phospho-tau signal extended beyond the two epitopes measured by Simoa (Fig. 4; Supplementary Tables 3 and 4). NULISA confirmed broad enrichment of tau-related proteins in newborns compared with aged controls. Plasma p-tau181 (mean log2FC = 4.40, 21.1-fold), p-tau217 (mean log2FC = 4.04, 16.5-fold), brain-derived MAPT (mean log2FC = 3.70, 13.0-fold), p-tau212 (mean log2FC = 2.56, 5.89-fold), p-tau231 (mean log2FC = 2.34, 5.07-fold) and p-tau205 (mean log2FC = 1.51, 2.85-fold) were all higher in newborns than in aged controls (BH-adjusted *P* < 0.001 for all). This pattern indicates that the neonatal tau signal was not restricted to p-tau217 and p-tau205 but instead involved multiple phosphorylated tau epitopes together with higher brain-derived MAPT.

**Figure 4:**
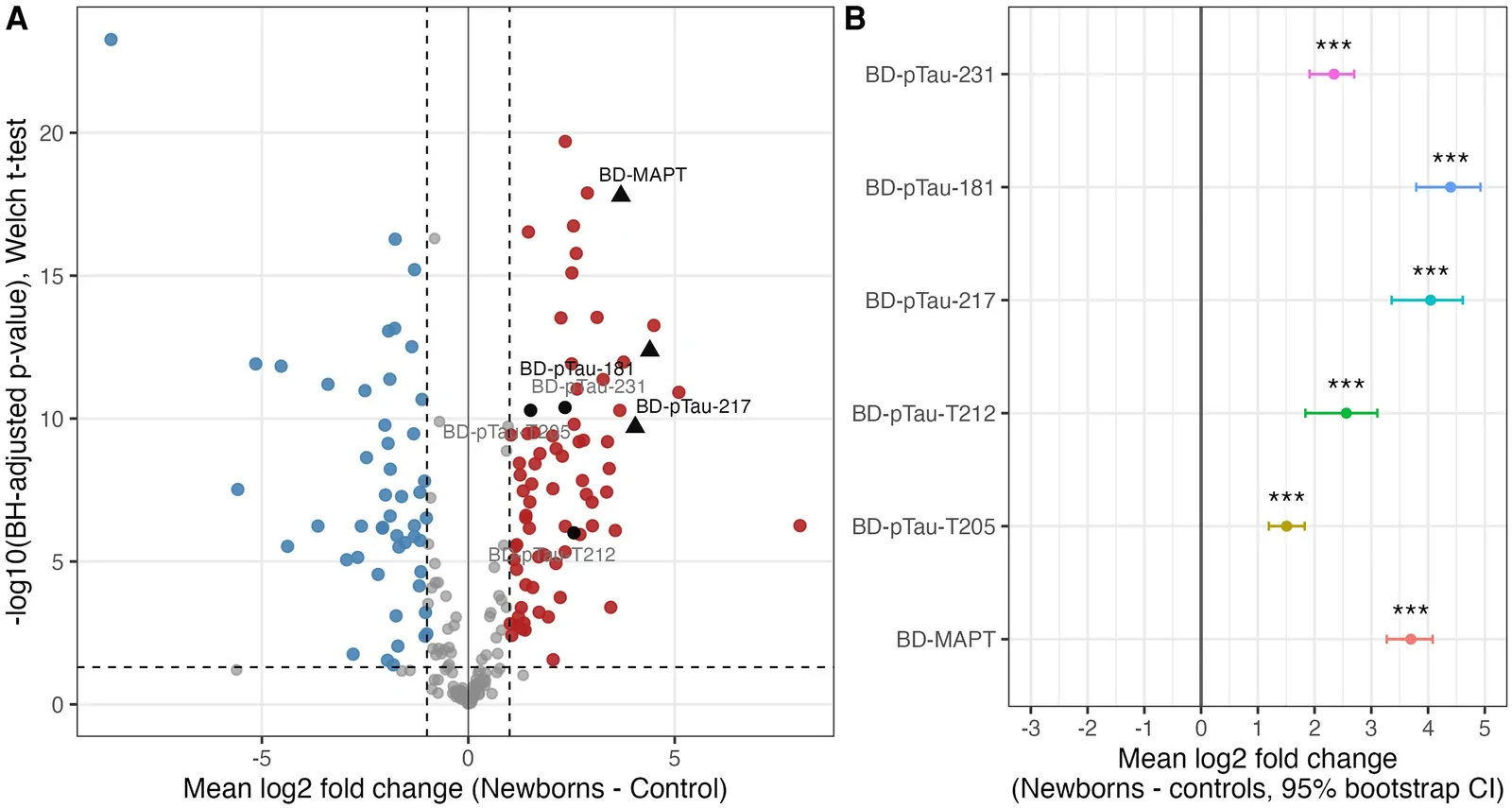
**NULISA CNS-panel analysis.** (Panel **A**) The volcano plot shows mean log2 fold change in Nucleic acid Linked Immuno-Sandwich Assay (NULISA) Protein Quantification (NPQ; newborns minus aged controls) on the *x*-axis and −log10 Benjamini–Hochberg (BH) adjusted Welch *t*-test *P*-values on the *y*-axis. Welch two-sample *t*-tests compared newborns (*n* = 27) with aged controls (*n* = 12), and each point represents one protein analyte. Positive log2FC values indicate higher protein levels in newborns, whereas negative values indicate lower levels in newborns. The dashed horizontal line indicates an adjusted *P*-value threshold of 0.05, and dashed vertical lines indicate the predefined absolute log2FC threshold (±1). Tau markers are highlighted and labelled, with top-ranked tau markers, defined by BH-adjusted significance followed by reciprocal fold-change magnitude (larger values indicating a greater fold increase or decrease), shown as black triangles with black labels, and remaining tau markers shown as black circles with grey labels. Non-tau proteins are included as background points. (Panel **B**) Forest plot showing mean log2 fold changes with 95% bootstrap confidence intervals for tau-related analytes in newborns (*n* = 27) compared with aged controls (*n* = 12); each point represents the mean log2 fold-change estimate for one tau-related analyte. Brain-derived (BD) p-tau181, p-tau217, p-tau231, p-tau212, p-tau205 and MAPT were all significantly higher in newborns than aged controls. The largest increases were observed for BD-pTau-181, BD-pTau-217, BD-pTau-231 and BD-MAPT, supporting a coordinated multi-epitope neonatal tau signature rather than an isolated elevation of a single phosphorylated tau species. Group differences were tested using Welch’s two-sample *t*-tests, with *P*-values adjusted for multiple testing across analytes using the Benjamini–Hochberg false discovery rate procedure. Asterisks denote statistically significant group differences based on BH-adjusted *P*-values (* adjusted *P* < 0.05; ** < 0.01; *** < 0.001). Abbreviations: CNS, central nervous system; log2FC, log2 fold change; BD, brain-derived; MAPT, microtubule-associated protein tau; p-tau, phosphorylated tau.

Importantly, the NULISA analysis also recapitulated the direction of the Simoa findings for glial, axonal and amyloid markers (Supplementary Table 4). GFAP was higher in newborns (mean log2FC = 1.01, 2.02-fold; BH-adjusted *P* < 0.01), whereas NEFL/NfL was lower (mean log2FC = −0.86, 1.81-fold lower; BH-adjusted *P* < 0.05). Amyloid species were also lower in newborns, with Aβ40 reduced 6.03-fold (mean log2FC = −2.59; BH-adjusted *P* < 0.001) and Aβ42 reduced 3.81-fold (mean log2FC = −1.93; BH-adjusted *P* < 0.001). Aβ38 was also numerically lower (1.66-fold lower), but this difference was not significant after multiple-testing correction (BH-adjusted *P* = 0.444).

Beyond standard Alzheimer’s disease biomarkers, the NULISA data highlighted a broader neonatal protein profile consistent with active neurodevelopmental and remodelling biology (Supplementary Fig. 2). Several neuronal, synaptic and cytoskeletal proteins were higher in newborns Supplementary Table 3, including NEFH (10.9-fold), NRGN (8.04-fold), GAP43 (7.39-fold), STMN2 (5.69-fold) and DLG4 (3.26-fold; BH-adjusted *P* < 0.001 for all). In contrast, several microglial, complement and inflammatory proteins were lower in newborns (Supplementary Table 4), including TREM2 (4.55-fold lower), CSF1R (2.88-fold lower), C1q (2.58-fold lower), CD33 (2.47-fold lower) and GRN (2.02-fold lower; BH-adjusted *P* < 0.001 for all). Overall, the NULISA results support the main Simoa findings by showing a multi-epitope neonatal phospho-tau enrichment, with moderate agreement between platforms (Supplementary Fig. 3), accompanied by high GFAP, low NfL, low amyloid species and a broader protein signature that differs from a typical Alzheimer’s disease-like injury profile.

## Discussion

In this study, we identify an early-life phospho-tau state that exceeds Alzheimer’s disease at multiple epitopes and is dissociated from an Alzheimer’s disease-like amyloid/neurodegeneration biomarker pattern. Our results show that phosphorylation of tau, a process central to both neuronal maturation and Alzheimer’s disease, is upregulated in healthy term human newborns across multiple epitopes, yet in a plasma biomarker context distinct from the amyloid and neurodegenerative profile typical of Alzheimer’s disease, consistent with foetal-tissue evidence that developmental phospho-tau can coexist with non-amyloid aggregates distinct from Alzheimer’s disease pathology.^34^

These findings might extend the concept of tau phosphorylation as a paradigm of antagonistic pleiotropy, in which a molecular programme that is beneficial during early life is later co-opted into neurodegenerative cascades.^8^ The same post-translational modifications that support axonal growth, synaptic refinement and circuit stabilization in the developing brain appear to become maladaptive in ageing, when they interact with chronic Aβ exposure, altered proteostasis and microglial dysregulation to drive tangle formation and neurodegeneration.^4,5,35^

A key observation is that both p-tau217 and p-tau205, classified within the 2024 AA framework as early (Core 1/T1) and later-stage (Core 2/T2) tau biomarkers, respectively, exhibit a striking stepwise increase from aged controls to Alzheimer’s disease and from Alzheimer’s disease to healthy term newborns in plasma.^13^ In adults, p-tau217 and p-tau205 track amyloid and tau pathology, and their elevation is interpreted as evidence of disease.^13,26,36^ In contrast, in neonates, these markers reach levels that exceed those seen in Alzheimer’s disease, despite the absence of amyloid deposition.^34^ Notably, the magnitude of the difference in p-tau between newborns and adults exceeds that between Alzheimer’s disease and controls, highlighting that the dynamic range of physiologic tau phosphorylation in early life is far greater than that typically observed along the Alzheimer’s disease continuum.^21,32^ One possible interpretation is that high phospho-tau states may have different biological significance in early and late life, but this hypothesis will require direct mechanistic testing.^8,37^ The exploratory NULISA CNS panel analysis strengthens this interpretation by showing that the neonatal tau signal is not restricted to the two Simoa-measured epitopes. In the newborn-control subset, multiple tau analytes, including phosphorylated tau epitopes such as p-tau181, p-tau205, p-tau212, p-tau217 and p-tau231, clustered among proteins with higher concentrations in newborns and strong statistical support. Although this exploratory subset analysis was smaller and should not be over-interpreted as a standalone discovery cohort, it provides independent multiplex evidence for broad early-life enrichment of tau biology and supports the view that neonatal phospho-tau elevation reflects a coordinated developmental state rather than an assay-specific artefact.^4,34^

Importantly, the neonatal phospho-tau profile is not merely a scaled-up version of the Alzheimer’s disease pattern. For instance, in Alzheimer’s disease, p-tau elevations co-occur with high NfL and rising GFAP, consistent with axonal and astroglial injury.^38^ In newborns, GFAP is indeed elevated, particularly after vaginal delivery, but NfL is low, and there is no evidence of the chronic axonal degeneration that characterizes Alzheimer’s disease.^39^ Furthermore, the mode-of-delivery analysis shows that while GFAP and NfL are highly sensitive to the mechanical and hypoxic challenges of vaginal birth, p-tau217 and p-tau205 are essentially insensitive to this perinatal stressor.^33^ This pattern is compatible with the possibility that neonatal p-tau elevations are not fully explained by acute birth-related injury, although it does not distinguish conclusively between developmental and other perinatal contributors.^40^

Our findings raise the possibility that the developing brain may possess unique mechanisms that allow it to tolerate, and indeed exploit, intense tau phosphorylation without progressing to Alzheimer’s disease-like tau pathology.^34^ Several non-mutually exclusive mechanisms can be proposed. First, isoform composition likely plays a critical role: the fetal and early postnatal human brain expresses distinct tau isoforms and splicing patterns, including a predominance of 0N3R tau, which may be inherently more soluble and less aggregation-prone than the mixed 3R/4R isoform landscape of the adult cortex.^11,41,42^ Second, the neonatal proteostasis network encompassing chaperones, ubiquitin, proteasome activity and autophagy is more robust than in ageing, potentially allowing rapid degradation of misfolded or excessively phosphorylated tau species before they can nucleate aggregates.^43,44^

In plasma, the associated biomarker pattern appears distinct from the typical Alzheimer’s disease profile and may reflect a different biological state; however, this interpretation should be made cautiously. Elevation of GFAP in newborns, particularly after vaginal delivery, may reflect astroglial activation that is adaptive rather than harmful, supporting synaptogenesis, metabolic buffering and extracellular matrix remodelling in response to physiological stressors.^39,45^ The modest or absent increase in NfL contrasts sharply with Alzheimer’s disease, where NfL is a robust indicator of large-calibre axonal degeneration.^33^

Additionally, our results could yield potential therapeutic implications. First, they caution strongly against a simplistic interpretation of p-tau elevations as inherently pathological.^46,47^ Any tau-targeting intervention, whether aiming to lower total tau, inhibit phosphorylation at specific epitopes, or enhance tau clearance, must take into account that some Alzheimer’s disease-associated phospho-tau epitopes are also present during development.^34,40,48,49^ This could have important implications for both drug development and safety assessment: aggressive life-long suppression of p-tau217 or p-tau205, for example, may have unforeseen consequences if administered during sensitive developmental windows or in younger at-risk populations.^19^ Second, the developmental context offers a template for ‘safe’ tau phosphorylation: understanding how neonatal neurons achieve high-level, multi-epitope phosphorylation without toxicity could reveal protective co-factors or network states that can be pharmacologically mimicked in the ageing brain.^5^ Enhancing chaperone systems, modulating specific kinase/phosphatase pairs or shifting the balance of tau isoforms towards more soluble species are all strategies that might draw inspiration from developmental biology.^21,50^

Finally, the present work does not call into question the value of p-tau biomarkers in Alzheimer’s disease; rather, it aims to underscore that the biology captured by ‘AD biomarkers’ is fundamentally pleiotropic: what signals degeneration in the aged brain is, in the newborn, a signature of growth, plasticity and adaptation.^2,3^ Understanding how this balance is lost with ageing may be key to altering the trajectory of tau-mediated neurodegeneration.

### Limitations

Several limitations should be taken into consideration for the interpretation of our findings. First, our study is cross-sectional and based on comparisons across independent cohorts, which precludes firm conclusions about developmental trajectories or causal mechanisms. Second, differences in biospecimen matrix, cohort composition and assay context further limit direct biological inference across groups. Accordingly, our results should be viewed primarily as evidence that high phospho-tau concentrations can occur in early life outside a typical Alzheimer-associated biochemical context, rather than as definitive proof of a specific developmental mechanism. Importantly, the NULISA analysis was exploratory, performed in 27 newborns and 12 adult comparator samples only, and did not include an Alzheimer’s disease comparator. Finally, we did not assess tau isoforms, kinase or phosphatase activity, proteostasis, neuroimaging, neuropathology or longitudinal developmental outcomes or APGAR scales. Longitudinal studies and analyses in independent neonatal cohorts will be needed to determine the extent to which these signals reflect physiological maturation, perinatal stress responses or disease-related processes.

## Supplementary Material

fcag413_Supplementary_Data

## Data Availability

Anonymized data are available on reasonable requests from the corresponding author. No custom software or analysis code was developed for this study.
