# Supplementary material for: The antagonistic pleiotropy of tau phosphorylation: plasma tau species in newborns and Alzheimer's disease
Source: Brain Commun. 2026 Oct 29;8(6):fcag413. doi: 10.1093/braincomms/fcag413 (PMC13748733; doi:10.1093/braincomms/fcag413)
Supplement: Supplementary file 1 [file fcag413_supplementary_data.pdf]

Supplementary appendix

The antagonistic pleiotropy of tau phosphorylation: Plasma tau species in newborns and Alzheimer's disease

Fernando Gonzalez-Ortiz, MD, PhD[1,2*]; Bjørn-Eivind Kirsebom, PhD[3,4,5*]; Cristiano Santos, PhD[1]; Burak Arslan, MD[1]; Julia K. Gundersen, MD, PhD[5]; Hlin Kvartsberg, PhD[6]; Karin Sävman, MD, PhD[7]; Michael Turton, PhD[8]; Peter Harrison, MA[8]; Helena Sophia Coley Gleerup, MD[9]; Kristian Steen Frederiksen, MD, PhD[9,10]; Steen Gregers Hasselbalch, MD, PhD[9,10]; Anja Hviid Simonsen, PhD[10]; Tormod Fladby, MD, PhD[5,11]; Helena Barreto Henriksson, PhD[12,13]; Sofia Frandberg, MD, PhD[13]; Ann Hellström, MD, PhD[12,14]; Floris Groenendaal, MD, PhD[15]; Michel N Nafash, MSc[16]; Ella N Klaus, MA[16]; Xuemei Zeng, PhD[16,17,18]; Thomas K. Karikari, PhD[16,17,18]; Henrik Zetterberg, MD, PhD[1, 6, 19,20,21,22,23]; Ulrika Sjöbom, PhD[12,14,24]

[*] Fernando Gonzalez-Ortiz and Bjørn-Eivind Kirsebom contributed equally to this work

Author affiliations:

1. Department of Psychiatry and Neurochemistry, Institute of Neuroscience and Physiology, the Sahlgrenska Academy at the University of Gothenburg, Mölndal, Sweden.
2. Royal College of Physicians of Edinburgh, Edinburgh, Scotland.
3. Department of Neurology, University Hospital of North Norway, Tromsø, Norway.
4. Department of Psychology, Faculty of Health Sciences, The Arctic University of Norway, Tromsø, Norway.
5. Department of Neurology, Akershus University Hospital, Lørenskog, Norway.
6. Clinical Neurochemistry Laboratory, Sahlgrenska University Hospital, Mölndal, Sweden.
7. Department of Paediatrics, Institution of Clinical Sciences, Sahlgrenska Academy, University of Gothenburg, Gothenburg, Sweden.
8. Bioventix Plc, 7 Romans Business Park, East Street, Farnham, Surrey GU9 7SX, UK.
9. Danish Dementia Research Centre, Department of Neurology, Copenhagen University Hospital, Rigshospitalet, Copenhagen, Denmark.
10. Department of Clinical Medicine, Faculty of Health and Medical Sciences, University of Copenhagen, Copenhagen, Denmark.
11. University of Oslo, Institute for Clinical Medicine, Campus Ahus, Oslo. Norway.

12. Department of Clinical Neuroscience at the Institution of Neuroscience and Physiology at Sahlgrenska Academy at University of Gothenburg, Gothenburg, Sweden.
13. Department of Laboratory Medicine, Institute of Biomedicine at Sahlgrenska Academy at University of Gothenburg, Gothenburg, Sweden.
14. Department of Ophthalmology, Sahlgrenska University Hospital, Region Västra Götaland, Gothenburg, Sweden.
15. Department of Neonatology, Wilhelmina Children's Hospital, University medical Center Utrecht and Utrecht University, Utrecht, Netherlands.
16. Department of Psychiatry, School of Medicine, University of Pittsburgh, Pittsburgh USA.
17. Biofluid Biomarker Laboratory, Western Psychiatric Hospital, University of Pittsburgh Medical Center, Pittsburgh, USA.
18. Alzheimer's Disease Research Center, University of Pittsburgh, Pittsburgh, Pennsylvania, USA.
19. Department of Neurodegenerative Disease, UCL Institute of Neurology, Queen Square, London, UK.
20. UK Dementia Research Institute at UCL, London, UK.
21. Department of Pathology and Laboratory Medicine, University of Wisconsin School of Medicine and Public Health, Madison, WI, USA.
22. Wisconsin Alzheimer's Disease Research Center, University of Wisconsin School of Medicine and Public Health, University of Wisconsin-Madison, Madison, WI, USA.
23. Centre for Brain Research, Indian Institute of Science, Bangalore, India.
24. Institute of Health and Care Sciences, Sahlgrenska Academy, University of Gothenburg, Gothenburg, Sweden.

Correspondence: Fernando Gonzalez-Ortiz, MD, PhD. Clinical Neurochemistry Lab House V3, floor 2 Mölndal Hospital Street Address: Biskopsbogatan 27 SE-43180 Mölndal, Sweden.

Email: fernando.gonzalez.ortiz@gu.se

Running title: Plasma tau species in newborns and AD

Keywords: Plasma phospho-tau; Babies; Alzheimer's disease; Neurodegeneration; Neurodevelopment

Supplementary appendix

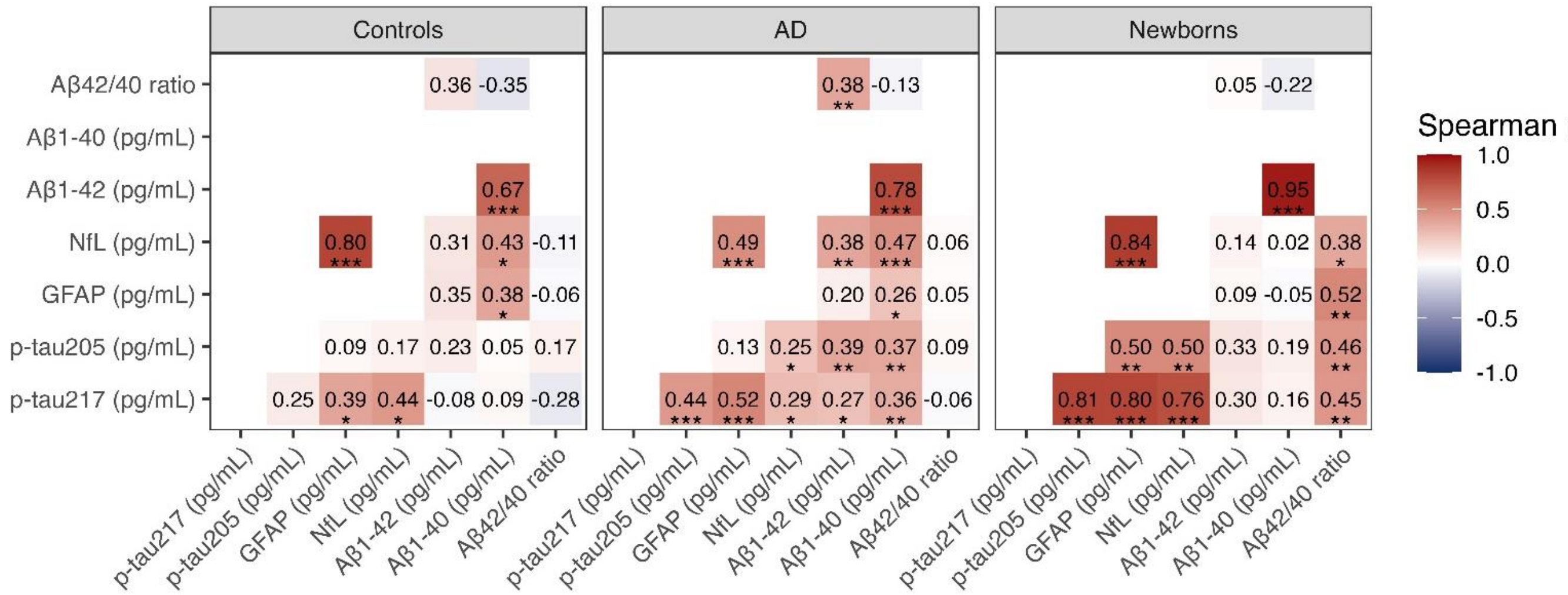

Supplementary Figure 1. Correlations between plasma AD-related biomarkers in adults with AD, cognitively unimpaired controls and newborns. Heatmaps display pairwise Spearman correlation coefficients (r) among plasma p-tau217, p-tau205, GFAP, NfL, Aβ1-42, Aβ1-40 and the Aβ42/40 ratio in controls (n = 41), individuals with AD (n = 73), and newborns (n = 42), respectively. The color scale encodes the magnitude and direction of r, from negative (blue) to positive (red) correlations. Numbers inside tiles indicate r, and asterisks denote the significance of the correlation as defined in the Methods.

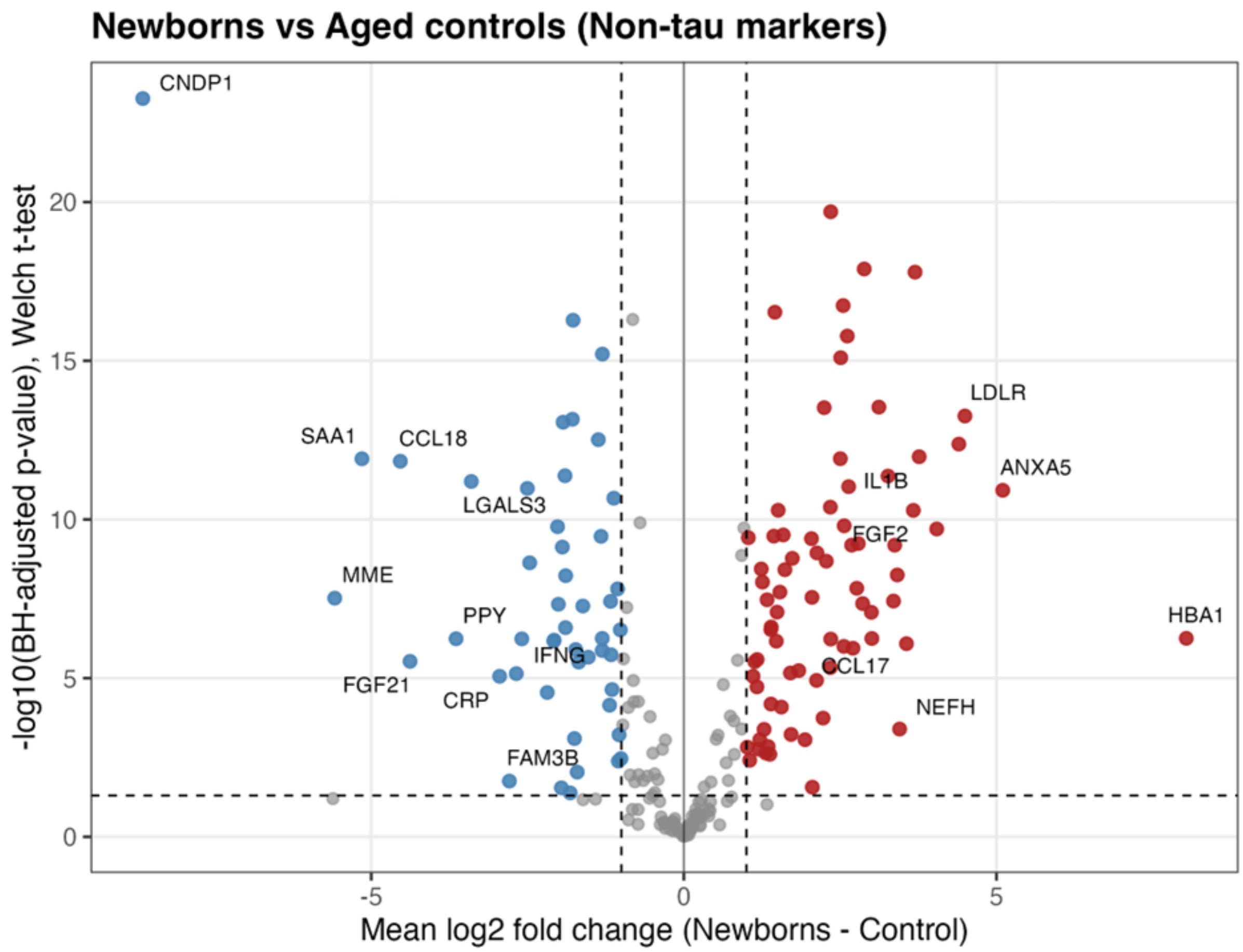

Supplementary Figure 2. Volcano plot of non-tau proteins in newborns (n = 27) compared with aged controls (n = 12). The volcano plot shows mean log2 fold change in NULISA Protein Quantification (NPQ; newborns minus aged controls) on the x-axis and −log10 Benjamini–Hochberg (BH) adjusted Welch t-test p-values on the y-axis. Positive log2FC values indicate higher protein levels in newborns, whereas negative values indicate lower levels in newborns. The dashed horizontal line indicates an adjusted p-value threshold of 0.05, and dashed vertical lines indicate the predefined absolute log2FC threshold (±1). Tau markers were excluded from this plot. Non-tau proteins are shown as points, with significant upregulated proteins shown in red, significant downregulated proteins shown in blue, and non-significant proteins shown in grey. Protein labels were assigned based on BH-adjusted significance followed by reciprocal fold-change magnitude (larger values indicating a greater fold increase or decrease).

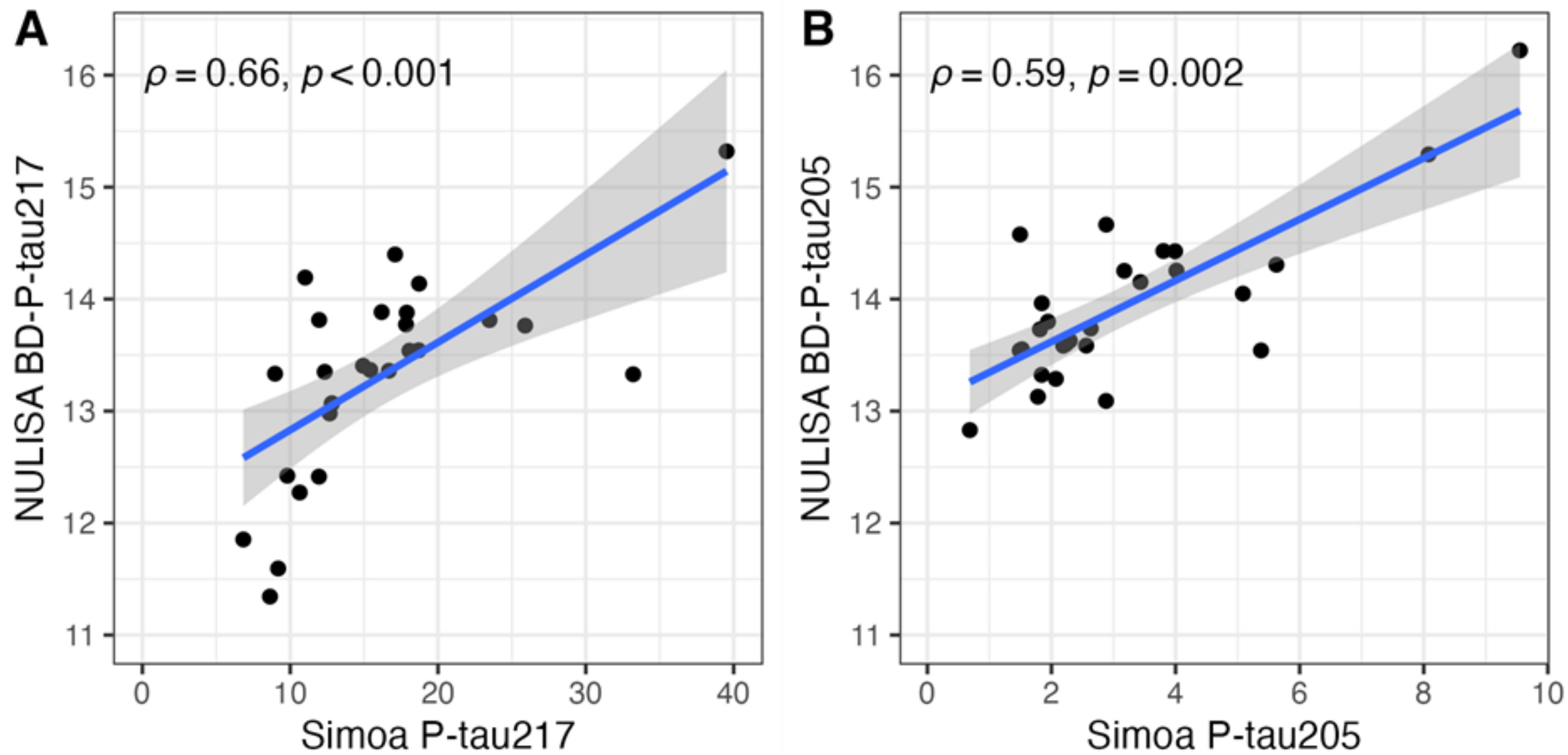

Supplementary Figure 3. Correlations between NULISA Brain-Derived tau markers and Quanterix HD-X Simoa p-tau measures in n = 27 newborns. Scatter plots showing Spearman's rank-order correlations between NULISA Brain-Derived (BD) p-tau217 and Quanterix HD-X Simoa p-tau217 results (**panel A**), and between NULISA Brain-Derived (BD) total (T) p-tau205 and Quanterix HD-X Simoa p-tau205 results (**panel B**). Each black dot represents an individual participant. Spearman's rank correlation coefficient (ρ) and the corresponding two-sided p-value are shown within each panel. Blue lines show the fitted linear regression line for visualization, with grey shaded areas indicating the 95% confidence interval.

Supplementary Table 1. Clinical characteristics of the NULISA newborn sub-cohort.

**Characteristic**	**NULISA sub-cohort (n = 27)**
Age, mean (SD), weeks	39.6 (0.9)
Female, n (%)	13 (48.1%)
Delivery mode, n (%)	
C-section	16 (59.3%)
Vaginal	11 (40.7%)
Birth weight, mean (SD), grams	3821.7 (403.4)

Notes. Values are presented as mean (SD) or n (%), as indicated. Twelve aged controls without clinical information were used as the comparison group for NULISA analyses. NULISA = Nucleic Acid-Linked Immuno-Sandwich Assay; SD = standard deviation.

Supplementary table 2. Paired Plasma Citrate and EDTA samples from n=8 controls from cohort 1.

Plasma biomarker	Mean (Citrate)	Mean (EDTA)	Mean ratio (EDTA/Citrate)	SD (EDTA/Citrate) ratio
p-tau217 (pg/mL)	1.55	3.63	2.33	0.68
p-tau205 (pg/mL)	1.34	0.83	0.93	0.56
NfL (pg/mL)	12.47	18.58	1.89	0.56
GFAP (pg/mL)	61.53	113.44	2.10	0.50
Aβ1-42 (pg/mL)	5.62	6.55	1.22	0.26
Aβ1-40 (pg/mL)	93.33	69.79	0.81	0.17

Notes. SD = standard deviation; pg/mL = picograms per milliliter; EDTA = ethylenediaminetetraacetic acid. The mean EDTA/citrate ratio was used as the correction factor for the Cohort 1 citrate plasma samples, which were subsequently compared with samples measured in EDTA plasma for the Simoa analysis.

Supplementary Table 3. Welch t-test comparisons of NULISA protein quantification differences between newborns and aged controls. This table shows proteins with higher levels in newborns compared with aged controls.

Analyte	Mean log2FC	Reciprocal fold change	95% CI	Welch t	p-value	BH-adjusted p
AGRN	1.73	3.33	1.4, 2.05	10.16	<0.001	<0.001
ANXA5	5.10	34.3	4.35, 5.87	12.38	<0.001	<0.001
APOE	0.19	1.14	-0.1, 0.46	1.29	0.212	0.253
AQP4	0.08	1.06	-0.86, 0.96	0.16	0.876	0.889
ATXN2	2.55	5.85	2.23, 2.88	15.18	<0.001	<0.001
ATXN3	2.12	4.35	1.38, 2.71	6.00	<0.001	<0.001
BACE1	1.03	2.04	0.8, 1.25	8.67	<0.001	<0.001
BCAN	1.17	2.25	0.73, 1.62	4.95	<0.001	<0.001
**BD-MAPT**	3.70	13.0	3.27, 4.08	17.68	<0.001	<0.001
**BD-pTau-181**	4.40	21.1	3.79, 4.92	14.33	<0.001	<0.001
**BD-pTau-217**	4.04	16.5	3.36, 4.61	12.15	<0.001	<0.001
**BD-pTau-231**	2.34	5.07	1.91, 2.7	11.13	<0.001	<0.001
**BD-pTau-T205**	1.51	2.85	1.2, 1.83	9.13	<0.001	<0.001
**BD-pTau-T212**	2.56	5.89	1.84, 3.11	7.66	<0.001	<0.001
CALB2	2.04	4.12	1.64, 2.45	9.67	<0.001	<0.001
CCL17	3.56	11.8	2.61, 4.36	7.57	<0.001	<0.001
CCL26	0.43	1.34	-0.04, 0.86	1.81	0.079	0.100
CCL4	0.11	1.08	-0.34, 0.53	0.47	0.642	0.667
CCL5	1.21	2.32	0.64, 1.79	3.94	<0.001	<0.01
CD40LG	3.27	9.62	2.73, 3.76	12.04	<0.001	<0.001
CD63	1.14	2.20	0.77, 1.52	5.66	<0.001	<0.001
CGRP	0.27	1.20	-0.15, 0.71	1.19	0.243	0.283
CNTN2	0.57	1.49	-0.65, 1.94	0.83	0.421	0.457
CRH	1.94	3.83	0.95, 2.96	3.63	<0.001	<0.01

Supplementary Table 3. Welch t-test comparisons of NULISA protein quantification differences between newborns and aged controls. This table shows proteins with higher levels in newborns compared with aged controls.

Analyte	Mean log2FC	Reciprocal fold change	95% CI	Welch t	p-value	BH-adjusted p
CST3	0.21	1.16	-0.07, 0.5	1.39	0.185	0.225
CTNNB1	2.35	5.09	2.12, 2.58	19.82	<0.001	<0.001
CXCL1	0.81	1.75	0.32, 1.25	3.32	<0.01	<0.01
CXCL8	0.26	1.20	-0.33, 0.86	0.85	0.403	0.444
DDC	0.05	1.03	-0.39, 0.44	0.21	0.834	0.850
DKK1	2.70	6.52	1.98, 3.26	7.98	<0.001	<0.001
DLG4	1.70	3.26	1.19, 2.23	6.20	<0.001	<0.001
DNM1L	2.68	6.43	2.22, 3.18	10.46	<0.001	<0.001
EIF4EBP1	2.35	5.10	1.69, 2.93	7.08	<0.001	<0.001
ENO2	1.54	2.90	1.18, 1.89	8.33	<0.001	<0.001
F2R	1.28	2.43	0.69, 1.73	4.56	<0.001	<0.001
FABP3	1.05	2.08	0.44, 1.61	3.33	<0.01	<0.01
FGF2	3.67	12.7	3.11, 4.14	13.34	<0.001	<0.001
FLT1	0.68	1.60	0.26, 1.09	3.08	<0.01	<0.01
FOLR1	0.14	1.10	-0.27, 0.51	0.71	0.488	0.517
GAP43	2.88	7.39	2.56, 3.2	17.26	<0.001	<0.001
GDF15	0.93	1.90	0.5, 1.33	4.28	<0.001	<0.001
GDI1	1.59	3.01	1.27, 1.88	9.69	<0.001	<0.001
GFAP	1.01	2.02	0.45, 1.54	3.49	<0.01	<0.01
GLP1R	0.11	1.08	-0.16, 0.38	0.77	0.452	0.489
HBA1	8.04	262	6.16, 9.41	9.00	<0.001	<0.001
HSPB1	3.00	7.99	2.47, 3.57	10.16	<0.001	<0.001
HTT	2.05	4.14	1.56, 2.49	8.42	<0.001	<0.001
IL10	0.39	1.31	-0.09, 0.87	1.54	0.134	0.167
IL13	1.20	2.30	0.59, 1.77	3.88	<0.01	<0.01
IL16	1.11	2.15	0.68, 1.52	5.22	<0.001	<0.001

Supplementary Table 3. Welch t-test comparisons of NULISA protein quantification differences between newborns and aged controls. This table shows proteins with higher levels in newborns compared with aged controls.

Analyte	Mean log2FC	Reciprocal fold change	95% CI	Welch t	p-value	BH-adjusted p
IL18	0.02	1.01	-0.55, 0.65	0.07	0.947	0.947
IL1B	3.76	13.6	3.09, 4.41	10.86	<0.001	<0.001
IL33	1.48	2.79	1.02, 1.9	6.22	<0.001	<0.001
IL4	1.49	2.81	1.1, 1.9	7.17	<0.001	<0.001
IL7	0.23	1.18	-0.16, 0.61	1.19	0.244	0.283
LDLR	4.50	22.6	3.8, 5.17	12.80	<0.001	<0.001
LRRK2	1.40	2.63	0.98, 1.82	6.31	<0.001	<0.001
MDH1	2.34	5.08	1.61, 3.01	6.33	<0.001	<0.001
MDK	0.63	1.55	0.41, 0.84	5.42	<0.001	<0.001
mHTT-exon1	0.76	1.70	0.09, 1.5	2.06	0.056	0.075
MMP10	0.10	1.07	-0.34, 0.48	0.45	0.654	0.672
MMP9	3.41	10.7	2.71, 4.07	9.48	<0.001	<0.001
NCAM1	0.24	1.18	0, 0.49	1.82	0.079	0.100
NEFH	3.45	10.9	1.96, 4.88	4.40	<0.001	<0.001
NFKB1	2.56	5.91	2.16, 2.95	11.86	<0.001	<0.001
NFKB2	2.61	6.12	2.32, 2.91	16.72	<0.001	<0.001
NPTX1	0.55	1.46	0.27, 0.82	3.77	<0.001	<0.01
NPTX2	0.22	1.17	-0.09, 0.56	1.31	0.202	0.243
NPY	0.69	1.62	-0.04, 1.41	1.84	0.076	0.098
NRGN	3.01	8.04	2.32, 3.73	8.18	<0.001	<0.001
NT-proBNP	1.33	2.51	-0.11, 2.68	1.78	0.095	0.120
Oligo-SNCA	2.06	4.16	0.28, 3.52	2.37	<0.05	<0.05
PAFAH1B3	1.38	2.60	0.57, 2.03	3.53	<0.01	<0.01
PARK7	2.64	6.22	2.25, 3.02	12.68	<0.001	<0.001
PARP1	0.92	1.90	0.74, 1.16	8.46	<0.001	<0.001
PGK1	3.37	10.4	2.69, 4.01	10.01	<0.001	<0.001

Supplementary Table 3. Welch t-test comparisons of NULISA protein quantification differences between newborns and aged controls. This table shows proteins with higher levels in newborns compared with aged controls.

Analyte	Mean log2FC	Reciprocal fold change	95% CI	Welch t	p-value	BH-adjusted p
pLRRK2-S1292	0.28	1.21	0.01, 0.55	1.96	0.066	0.086
POSTN	2.28	4.85	1.85, 2.73	9.75	<0.001	<0.001
PPBP	0.52	1.43	0.25, 0.77	3.75	<0.001	<0.01
pPRKN-S65	0.43	1.35	0.1, 0.77	2.47	<0.05	<0.05
pQ-ATXN3	1.35	2.55	0.61, 1.96	3.73	<0.01	<0.01
pQ-HTT	1.33	2.52	0.98, 1.69	7.15	<0.001	<0.001
pRAB10-T73	0.96	1.95	0.76, 1.18	8.81	<0.001	<0.001
pRAB12-S106	0.86	1.81	0.57, 1.14	5.64	<0.001	<0.001
PRDX5	2.24	4.74	1.89, 2.61	11.98	<0.001	<0.001
PRDX6	2.86	7.26	2.21, 3.4	9.11	<0.001	<0.001
PRKN	0.80	1.74	0.44, 1.13	4.29	<0.001	<0.001
PSEN1	1.24	2.36	0.93, 1.53	8.00	<0.001	<0.001
PSME1	0.34	1.27	-0.15, 0.77	1.38	0.188	0.228
pSNCA-129	2.23	4.68	1.19, 3.08	4.44	<0.001	<0.001
pTDP43-409	1.44	2.71	1.16, 1.77	8.87	<0.001	<0.001
PTPRS	0.33	1.25	0.06, 0.59	2.37	<0.05	<0.05
RAB10	2.50	5.67	2.05, 2.96	10.75	<0.001	<0.001
RAB12	1.84	3.57	1.28, 2.36	6.47	<0.001	<0.001
RAB29	0.19	1.14	-0.03, 0.44	1.53	0.135	0.168
REST	1.26	2.39	0.95, 1.59	7.38	<0.001	<0.001
RUVBL2	1.62	3.07	1.25, 1.96	8.55	<0.001	<0.001
S100A12	2.79	6.92	2.15, 3.41	8.46	<0.001	<0.001
S100B	1.17	2.25	0.79, 1.53	5.95	<0.001	<0.001
SFRP1	0.71	1.64	0.17, 1.23	2.58	<0.05	<0.05
SMOC1	1.72	3.28	0.86, 2.59	3.80	<0.001	<0.01
SNCA	1.31	2.48	0.56, 1.94	3.53	<0.01	<0.01

Supplementary Table 3. Welch t-test comparisons of NULISA protein quantification differences between newborns and aged controls. This table shows proteins with higher levels in newborns compared with aged controls.

Analyte	Mean log2FC	Reciprocal fold change	95% CI	Welch t	p-value	BH-adjusted p
SNCB	0.08	1.06	-0.13, 0.28	0.75	0.460	0.492
SOD1	1.56	2.95	0.93, 2.09	5.08	<0.001	<0.001
SPP1	3.35	10.2	2.69, 3.88	10.77	<0.001	<0.001
SQSTM1	1.39	2.63	0.88, 1.87	5.23	<0.001	<0.001
STMN2	2.51	5.69	2.17, 2.83	14.61	<0.001	<0.001
STX4	2.13	4.37	1.73, 2.51	10.50	<0.001	<0.001
TARDBP	3.12	8.69	2.65, 3.6	12.59	<0.001	<0.001
TIMP3	2.77	6.80	2.04, 3.46	7.40	<0.001	<0.001
TNFSF14	0.40	1.32	-0.21, 0.96	1.27	0.219	0.261
TREM1	0.42	1.34	-0.12, 0.91	1.51	0.150	0.183
UCHL1	0.18	1.13	-0.09, 0.5	1.20	0.241	0.282
VCAM1	0.13	1.10	-0.08, 0.33	1.25	0.225	0.265
VEGFA	0.74	1.67	0.41, 1.02	4.64	<0.001	<0.001
VGF	0.26	1.20	-0.39, 0.95	0.75	0.456	0.490
VSNL1	1.39	2.62	1.01, 1.74	7.07	<0.001	<0.001
YWHAG	1.46	2.74	1.28, 1.64	15.46	<0.001	<0.001
YWHAZ	0.08	1.06	-0.11, 0.24	0.82	0.418	0.456

Notes. NULISA = Nucleic Acid-Linked Immuno-Sandwich Assay; NPQ = NULISA Protein Quantification; log2FC = mean log2 fold change, calculated as mean NPQ in newborns minus mean NPQ in aged controls. Reciprocal fold change is shown to aid interpretation: for proteins higher in newborns, this corresponds to the fold increase in newborns relative to aged controls, whereas for proteins lower in newborns, it corresponds to the fold decrease in newborns relative to aged controls. Confidence intervals are bootstrap 95% confidence intervals for the mean log2FC. P-values are from Welch's two-sample t-tests and were adjusted across analytes using the Benjamini–Hochberg (BH) procedure; both raw and BH-adjusted p-values are shown. Tau proteins are highlighted in bold. *Proteins are ordered alphabetically.*

Supplementary Table 4. Welch t-test comparisons of NULISA protein quantification differences between newborns and aged controls. This table shows proteins with lower levels in newborns compared with aged controls.

Analyte	Mean log2FC	Reciprocal fold change	95% CI	Welch t	p-value	BH-adjusted p
ACHE	-0.81	1.75	-1.09, -0.52	-5.40	<0.001	<0.001
APOA1	-0.70	1.62	-0.81, -0.59	-12.04	<0.001	<0.001
APOA2	-0.82	1.76	-0.92, -0.73	-15.56	<0.001	<0.001
APOE4	-5.61	49.0	-10.39, -1.59	-2.26	0.062	0.082
APOH	-1.89	3.71	-2.34, -1.47	-8.31	<0.001	<0.001
ARSA	-1.19	2.28	-1.62, -0.77	-5.29	<0.001	<0.001
AXL	-0.15	1.11	-0.49, 0.15	-0.91	0.372	0.417
Aβ38	-0.73	1.66	-2.07, 0.63	-0.93	0.402	0.444
Aβ40	-2.59	6.03	-3.34, -1.85	-6.53	<0.001	<0.001
Aβ42	-1.93	3.81	-2.26, -1.62	-11.51	<0.001	<0.001
BASP1	-1.01	2.01	-1.56, -0.43	-3.23	<0.01	<0.01
BDNF	-0.22	1.17	-1.01, 0.42	-0.59	0.567	0.595
C1q	-1.37	2.58	-1.57, -1.17	-12.92	<0.001	<0.001
CCL11	-2.08	4.23	-2.59, -1.62	-7.83	<0.001	<0.001
CCL13	-0.72	1.65	-1.22, -0.27	-2.84	<0.05	<0.05
CCL18	-4.53	23.2	-5.15, -3.91	-13.77	<0.001	<0.001
CCL2	-1.68	3.21	-2.15, -1.21	-6.64	<0.001	<0.001
CCL22	-0.65	1.57	-1.09, -0.21	-2.70	<0.05	<0.05
CCL3	-0.50	1.41	-0.97, -0.09	-2.13	<0.05	0.067
CD33	-1.31	2.47	-1.75, -0.88	-5.79	<0.001	<0.001
CHI3L1	-0.31	1.24	-0.95, 0.34	-0.91	0.378	0.421
CHIT1	-0.38	1.30	-1.2, 0.46	-0.85	0.409	0.449
CNDP1	-8.65	403	-9.18, -8.16	-32.48	<0.001	<0.001
CRP	-2.95	7.71	-3.69, -2.23	-7.54	<0.001	<0.001
CSF1R	-1.53	2.88	-1.98, -1.06	-6.14	<0.001	<0.001
CSF2	-0.58	1.50	-0.97, -0.19	-2.77	<0.05	<0.05

Supplementary Table 4. Welch t-test comparisons of NULISA protein quantification differences between newborns and aged controls. This table shows proteins with lower levels in newborns compared with aged controls.

Analyte	Mean log2FC	Reciprocal fold change	95% CI	Welch t	p-value	BH-adjusted p
CST5	-0.73	1.66	-1.67, 0.17	-1.52	0.138	0.170
CX3CL1	-0.54	1.45	-0.77, -0.31	-4.42	<0.001	<0.001
CXCL10	-2.01	4.02	-2.43, -1.56	-8.61	<0.001	<0.001
CXCL13	-0.46	1.38	-0.87, -0.05	-2.16	<0.05	0.055
EDA2R	-1.82	3.53	-3.42, -0.31	-2.22	<0.05	0.056
EFEMP1	-1.77	3.41	-1.99, -1.56	-15.52	<0.001	<0.001
EGFR	-1.78	3.44	-2.04, -1.53	-13.34	<0.001	<0.001
FAM3B	-2.79	6.92	-4.9, -0.97	-2.76	<0.05	<0.05
FCGR2A	-1.41	2.66	-2.69, -0.05	-2.00	0.065	0.085
FCGR2B	-0.88	1.85	-2.6, 0.38	-1.11	0.292	0.334
FCN2	-2.50	5.67	-2.88, -2.15	-13.04	<0.001	<0.001
FGF21	-4.38	20.8	-5.5, -3.16	-7.07	<0.001	<0.001
GAS6	-1.73	3.32	-2.23, -1.24	-6.75	<0.001	<0.001
GBA	-0.30	1.23	-0.45, -0.13	-3.62	<0.001	<0.01
GDNF	-0.30	1.23	-1.21, 0.63	-0.62	0.540	0.569
GOT1	-0.17	1.12	-0.49, 0.15	-1.00	0.328	0.373
GPNMB	-1.32	2.50	-1.62, -1.06	-9.05	<0.001	<0.001
GRN	-1.02	2.02	-1.28, -0.75	-7.24	<0.001	<0.001
GSDMD	-1.61	3.06	-3.29, -0.38	-2.02	0.068	0.088
GSDME	-0.18	1.14	-0.54, 0.17	-0.97	0.340	0.383
HMOX1	-1.16	2.24	-1.5, -0.82	-6.63	<0.001	<0.001
HPGDS	-0.80	1.74	-1.12, -0.47	-4.56	<0.001	<0.001
ICAM1	-1.30	2.47	-1.67, -0.94	-6.85	<0.001	<0.001
IFNG	-2.68	6.41	-3.56, -1.95	-6.30	<0.001	<0.001
IGF1R	-0.34	1.27	-0.53, -0.17	-3.55	<0.01	<0.01
IGFBP7	-1.17	2.25	-1.44, -0.93	-8.80	<0.001	<0.001

Supplementary Table 4. Welch t-test comparisons of NULISA protein quantification differences between newborns and aged controls. This table shows proteins with lower levels in newborns compared with aged controls.

Analyte	Mean log2FC	Reciprocal fold change	95% CI	Welch t	p-value	BH-adjusted p
IL12p70	-1.62	3.06	-2.01, -1.26	-8.19	<0.001	<0.001
IL15	-1.06	2.08	-1.33, -0.81	-7.77	<0.001	<0.001
IL17A	-1.75	3.36	-2.57, -1.04	-4.33	<0.001	<0.01
IL2	-0.98	1.97	-1.4, -0.58	-4.47	<0.001	<0.001
IL5	-1.04	2.05	-1.53, -0.54	-3.92	<0.001	<0.01
IL6	-0.17	1.13	-0.91, 0.53	-0.46	0.651	0.672
IL6R	-0.97	1.95	-1.29, -0.65	-5.80	<0.001	<0.001
IL9	-1.05	2.07	-1.64, -0.45	-3.30	<0.01	<0.01
ITGAV	-2.47	5.52	-2.85, -2.1	-12.41	<0.001	<0.001
KDR	-0.91	1.88	-1.17, -0.66	-6.78	<0.001	<0.001
KLK6	-0.12	1.09	-0.46, 0.19	-0.73	0.472	0.503
L1CAM	-1.89	3.71	-2.34, -1.5	-8.60	<0.001	<0.001
LGALS3	-3.40	10.6	-3.97, -2.81	-11.14	<0.001	<0.001
MAG	-0.08	1.06	-0.38, 0.37	-0.39	0.706	0.723
MME	-5.58	48.0	-6.82, -4.4	-8.79	<0.001	<0.001
MOG	-1.15	2.22	-1.62, -0.72	-4.84	<0.001	<0.001
MSLN	-0.78	1.72	-1.35, -0.21	-2.61	<0.05	<0.05
NEFL	-0.86	1.81	-1.44, -0.31	-2.89	<0.05	<0.05
NELL1	-2.07	4.21	-2.56, -1.58	-7.72	<0.001	<0.001
NGF	-0.36	1.29	-0.98, 0.07	-1.24	0.236	0.278
NLRP3	-1.71	3.26	-2.72, -0.71	-3.13	<0.01	<0.05
NPTXR	-0.47	1.38	-0.79, -0.14	-2.71	<0.05	<0.05
NTRK2	-1.12	2.17	-1.33, -0.91	-10.22	<0.001	<0.001
NTRK3	-0.49	1.41	-0.76, -0.22	-3.50	<0.01	<0.01
PDGFC	-0.09	1.07	-0.43, 0.2	-0.57	0.571	0.596
PDGFRB	-0.55	1.46	-1.04, -0.02	-2.02	0.061	0.081

Supplementary Table 4. Welch t-test comparisons of NULISA protein quantification differences between newborns and aged controls. This table shows proteins with lower levels in newborns compared with aged controls.

Analyte	Mean log2FC	Reciprocal fold change	95% CI	Welch t	p-value	BH-adjusted p
PDLIM5	-1.96	3.89	-3.51, -0.56	-2.50	<0.05	<0.05
PGF	-1.30	2.47	-1.48, -1.12	-14.10	<0.001	<0.001
PLAUR	-0.39	1.31	-0.8, -0.03	-1.90	0.077	0.099
PPY	-3.64	12.5	-4.5, -2.8	-8.08	<0.001	<0.001
pRAB29-T71	-0.01	1.01	-0.3, 0.29	-0.07	0.943	0.947
PROS1	-1.90	3.73	-2.1, -1.69	-17.82	<0.001	<0.001
PTN	-0.32	1.25	-0.98, 0.2	-0.99	0.340	0.383
SAA1	-5.15	35.5	-6.09, -4.21	-10.49	<0.001	<0.001
SERPINA3	-2.02	4.06	-2.33, -1.74	-12.63	<0.001	<0.001
SFTPD	-1.94	3.84	-2.31, -1.56	-9.69	<0.001	<0.001
SLIT2	-0.73	1.66	-1.01, -0.47	-5.12	<0.001	<0.001
SNAP25	-0.01	1.00	-0.1, 0.08	-0.11	0.915	0.924
TEK	-0.14	1.10	-0.39, 0.09	-1.13	0.267	0.307
TNF	-0.41	1.33	-0.71, -0.13	-2.69	<0.05	<0.05
TREM2	-2.18	4.55	-2.88, -1.56	-6.25	<0.001	<0.001
UBB	-0.82	1.77	-1.89, 0.1	-1.57	0.137	0.169
VEGFD	-0.89	1.85	-1.22, -0.57	-5.16	<0.001	<0.001

Notes. NULISA = Nucleic Acid-Linked Immuno-Sandwich Assay; NPQ = NULISA Protein Quantification; log2FC = mean log2 fold change, calculated as mean NPQ in newborns minus mean NPQ in aged controls. Reciprocal fold change is shown to aid interpretation: for proteins higher in newborns, this corresponds to the fold increase in newborns relative to aged controls, whereas for proteins lower in newborns, it corresponds to the fold decrease in newborns relative to aged controls. Confidence intervals are bootstrap 95% confidence intervals for the mean log2FC. P-values are from Welch's two-sample t-tests and were adjusted across analytes using the Benjamini–Hochberg (BH) procedure; both raw and BH-adjusted p-values are shown. *Proteins are ordered alphabetically.*
